# Anti-citrullinated fibronectin antibodies in rheumatoid arthritis are associated with human leukocyte antigen-*DRB1 *shared epitope alleles

**DOI:** 10.1186/ar3744

**Published:** 2012-02-17

**Authors:** Joyce JBC van Beers, Annemiek Willemze, Judith Stammen-Vogelzangs, Jan W Drijfhout, René EM Toes, Ger J M Pruijn

**Affiliations:** 1 Department of Biomolecular Chemistry, Institute for Molecules and Materials, Nijmegen Center for Molecular Life Sciences, Radboud University Nijmegen, P.O. Box 9101, 6500 HB Nijmegen, The Netherlands; 2 Department of Rheumatology, Leiden University Medical Center, P.O. Box 9600, 2300 RC Leiden, The Netherlands; 3 Department of Immunohematology and Blood Transfusion, Leiden University Medical Center, P.O. Box 9600, 2300 RC Leiden, The Netherlands

**Keywords:** rheumatoid arthritis, fibronectin, autoantigen, citrullination, ACPA

## Abstract

**Introduction:**

Fibronectin is one of the most abundant proteins present in the inflamed joint. Here, we characterized the citrullination of fibronectin in the joints of rheumatoid arthritis (RA) patients and studied the prevalence, epitope specificity and human leukocyte antigen (HLA) association of autoantibodies against citrullinated fibronectin in RA.

**Methods:**

Citrullinated residues in fibronectin isolated from RA patient synovial fluid were identified by mass spectrometry. The corresponding citrullinated and non-citrullinated peptides were synthesized and used to analyze the presence of autoantibodies to these peptides in RA sera and sera from other diseases and healthy controls by ELISA. The data were compared with risk factors like shared epitope HLA alleles and smoking, and with clinical features.

**Results:**

Five citrullinated residues were identified in fibronectin from RA synovial fluid. RA sera reacted in a citrulline-dependent manner with two out of four citrullinated fibronectin peptides, one of which contains two adjacent citrulline residues, in contrast to non-RA sera, which were not reactive. The most frequently recognized peptide (FN-Cit_1035,1036_, LTVGLTXXGQPRQY, in which × represents citrulline) was primarily targeted by anti-CCP (cyclic citrullinated peptide) 2-positive RA patients. Anti-FN-Cit_1035,1036 _autoantibodies were detected in 50% of established anti-CCP2-positive RA patients and in 45% of such patients from a early arthritis clinic. These antibodies appeared to be predominantly of the immunoglobulin G (IgG) isotype and to be associated with HLA shared epitope alleles (odds ratio = 2.11).

**Conclusions:**

Fibronectin in the inflamed synovia of RA patients can be citrullinated at least at five positions. Together with the flanking amino acids, three of these citrullinated residues comprise two epitopes recognized by RA autoantibodies. Anti-citrullinated fibronectin peptide antibodies are associated with HLA shared epitope alleles.

## Introduction

Citrullination or deimination is a post-translational modification, in which a peptidylarginine is converted into a peptidylcitrulline by the enzyme family of peptidylarginine deiminases (PAD). Citrullinated proteins occur at inflamed sites in healthy individuals as well as in patients [1,2]. However, autoantibodies directed against citrullinated proteins (anti-citrullinated protein/peptide antibodies, ACPA) are very specific for rheumatoid arthritis (RA). More than 70% of RA patients display ACPA, measured via the anti-CCP2 (cyclic citrullinated peptide 2) test, in their sera [3,4]. These antibodies are frequently present prior to disease onset and can predict the development of RA [5,6].

It is still not fully understood how RA originates and develops, although there is experimental evidence for several steps in this process [7]. Both genetic and environmental factors have been demonstrated to contribute to the development of the disease and ACPA production. The association of several *HLA-DRB1 *alleles, which all share a highly conserved motif that is known as the shared epitope (SE), has already been reported many years ago [8,9]. Other genes that have been identified as risk factors for RA include *PTPN22*, the *TRAF1-C5 *locus*, PADI4, STAT4, IRF5 *and *CTLA-4 *[10-15]. Smoking has been demonstrated to be an environmental risk factor for RA and also for ACPA production in RA patients carrying SE alleles [16,17]. Other environmental risk factors that have been suggested to enhance the chance of developing RA include the exposure to mineral oil, diet restrictions and coffee intake [18-20]. However, these data still need confirmation.

Many citrullinated autoantigens (for example, fibrinogen, vimentin, α-enolase) and ACPA directed towards these citrullinated proteins have been identified in RA [21-26]. Currently, the CCP2 test, which is based on a synthetic citrullinated peptide not related to proteins occurring in the inflamed joints of RA patients, is the gold standard [27-29] for ACPA testing. ACPA have recently been included in the new American College of Rheumatology/European League Against Rheumatism (ACR/EULAR) criteria for the classification of RA, because they are present early in the disease and can predict disease development and outcome [5,30]. The ACPA response in established RA patients is very heterogeneous and includes antibodies directed to many citrullinated proteins [31-33]. Because it has been suggested that ACPA play an important role in the development of the disease, it is important to learn more about the autoantigens that could be involved in the generation of ACPA [7]. Several citrullinated proteins occurring in the inflamed joints of RA patients have been identified previously. In part, their citrulline-containing epitopes have been mapped, particularly using synthetic peptides [24,34] or material from cultured (non-synovial) cells (for example, HL-60 cells) [23]. It remains to be established whether these epitopes are relevant from a pathophysiological point of view. ACPA tests based upon citrullinated autoantigenic proteins may provide information on ACPA fine-specificities [35,36] and may aid the differentiation between clinically distinct RA patient subgroups, although so far no correlations between ACPA fine-specificities and clinical phenotypes have been found [37-39].

One of the citrullinated proteins in inflamed synovial tissue identified previously is fibronectin (FN) [40,41]. FN is a glycoprotein, which can be a component of the extracellular matrix (insoluble form) or present in body fluids (soluble form). FN is involved in a variety of processes, such as wound healing, haemostasis, thrombosis and embryogenesis [42]. Several findings have been published linking (citrullinated) FN with RA. For example, citrullinated FN was found to be present in synovial tissue and synovial fluid (SF) of RA patients [41,43]. It has also been detected in pannus tissue and in immune complexes present in sera of RA patients [44,45]. FN might play a role in articular cartilage destruction, because it has been observed that FN fragments can stimulate the production of multiple mediators of matrix destruction, such as various cytokines and metalloproteinases [46,47]. However, the presence and characteristics of anti-citrullinated fibronectin antibodies in RA patients have not been studied yet.

In the current study, we have mapped citrullinated residues of FN isolated from the SF of RA patients and have used this information to investigate the B-cell response to citrullinated FN in RA.

## Materials and methods

### Patient material

Synovial fluid (SF) samples from RA patients were a kind gift from Prof. Dr. B. Bozic (Department of Rheumatology, University Medical Center, Ljubljana, Slovenia). After SF samples were obtained by joint punctures (arthrocentesis), using needles with a diameter from 1.6 to 2.2 mm, they were immediately centrifuged at 2,500 × g for 10 minutes at 4°C to separate insoluble and soluble components into pellet and supernatant fractions, respectively. Supernatant and pellet fractions were separately stored at -80°C within two hours after taking the samples. The pellets were resuspended in EGTA lysis buffer (50 mM Tris-HCl, pH 7.4, 100 mM KCl, 1 mM DTE, 0.1% NP40, 10 mM EGTA, 0.5 mM PMSF and protease inhibitor cocktail). Supernatant fractions were diluted in five volumes of EGTA lysis buffer. After sonification, SDS was added (final concentration 2%) and the fractions were heated and centrifuged at 12,000 × g. Supernatants were used for further analysis.

Sera from (established) rheumatoid arthritis (RA; *n *= 110), systemic lupus erythematosus (SLE; *n *= 31), and primary Sjőgren's syndrome (pSS; *n *= 31) patients were collected at the Department of Rheumatology of the University Medical Centre Nijmegen and the St. Maartenskliniek Nijmegen (The Netherlands). Sera from multiple sclerosis (MS; *n *= 31) patients were collected at the MS Centrum Nijmegen (The Netherlands). Type 1 diabetes (T1D; *n *= 32) sera were obtained from the Department of Immunohaematology and Blood Transfusion of the Leiden University Medical Centre (Leiden, The Netherlands). Early arthritis sera (EAC; *n *= 301) were collected at the Department of Rheumatology of the Leiden University Medical Center (Leiden, The Netherlands) [48]. Tuberculosis (TB; *n *= 29) sera were collected at Department of Internal Medicine, Tel Aviv Medical Center, Israel. Sera from healthy individuals (NS; *n *= 32) were collected at the Sanquin Blood Bank in Nijmegen. Sera were stored at -70°C until use.

ACPA levels in RA sera were measured using a commercial CCP2 ELISA kit (Euro-Diagnostica A.B., Malmo, Sweden).

These studies were approved by the local ethics committees; the need for patient consent was waived by the local ethics committees.

### Sample preparation and tandem mass spectrometry analysis

Two SF samples from RA patients, the supernatant fraction of one and the pellet of the other, were depleted of albumin as described by Colantonio and coworkers [49], separated by SDS-PAGE and stained with colloidal Coomassie Brilliant Blue. Each lane of the gel, containing material from an individual SF sample, was sliced into 18 pieces and the polypeptides in these gel slices were digested after the addition of 20 μL trypsin solution (15 ng/μL trypsin in 25 mM NH_4_HCO_3 _and 5 mM n-octylpyranoglucoside). Peptides were extracted by adding 50% acetonitril, 0.5% trifluoroacetic acid, 5 mM n-octylpyranoglucoside followed by sonication. The protein digests resulting from each of the gel slices were separately analyzed by nano-LC-MS/MS (using a LTQ (linear trap quadrupole) Fourier Transform Ion Cyclotron Resonance mass spectrometer (LTQ FT, Thermo Scientific, Waltham, MA, USA)). Data were converted by BioWorks SEQUEST (Thermo Electron, Waltham, MA, USA) into a peak list, which allowed peptide identification with the Mascot Search database. Additionally, citrullination sites were checked manually. Mass deviations for precursor ions were set to 20 ppm and deviations for the mass of fragment ions were set at 0.8 Da. Fixed modifications, besides citrullination, such as oxidation and methylation, were taken along during the analysis.

### Synthesis of citrullinated fibronectin peptides

Peptides (Table 1) were synthesized by a solid-phase procedure using Fmoc chemistry as described previously [50]. The peptides were at least 90% pure as deduced from their elution pattern on reversed phase HPLC.

**Table 1:** Sequences of synthetic fibronectin peptides

Peptide name	Peptides identified in RA SF^a^	Synthetic peptide^a^	Citrulline residues identified in RA1	Citrulline residues identified in RA2
FN-Cit_241_	DTRTSY**X**IGDTWSK, TSY**X**IGDTWSK	DTRTSY**X**IGDTWSZO	X	X
FN-Arg_241_		DTRTSYRIGDTWSZO		
FN-Cit_1035,1036_	LTVGLT**XX**GQPR, AQITGYRLTVGLT**X**R	LTVGLT**XX**GQPRQYZO	X	X^b^
FN-Arg_1035,1036_		LTVGLTRRGQPRQYZO		
FN-Cit_1162_	DGQE**X**DAPIVNK	LRDGQE**X**DAPIVNZO	X	X
FN-Arg_1162_		LRDGQERDAPIVNZO		
FN-Cit_2356_	RPGGEPSPEGTTGQSYNQYSQ**X**YHQR	YNQYSQ**X**YHQRTNZO		X
FN-Arg_2356_		YNQYSQRYHQRTNZO		

^a ^O, biotinylated lysine; X, citrulline; Z, aminohexanoic acid. ^b ^Only citrulline at position 1035 was identified

### Enzyme-linked immunosorbent assay

Enzyme-linked immunosorbent assays (ELISA) with fibronectin peptides were performed as described previously. Briefly, each well of a microtiter plate (Streptawell, Roche, Basel, Switzerland) was coated with 1 μg biotinylated peptide in 0.1 mL PBS/0.1% BSA overnight at 4°C. After washing three times with PBST_0.1 _(PBS, 0.1% Tween20), wells were incubated with serum samples 100-fold diluted at in PBST_0.05 _(PBS, 0.05% Tween20) containing 1% BSA for one hour at 37°C. After incubation, plates were washed three times with PBST_0.1_, followed by an incubation at 37°C with HRP-conjugated goat anti-human IgG, IgM, IgA, kappa, lambda (DAKO, Glostrup, Denmark) or with either HRP-conjugated rabbit anti-human IgG, rabbit anti-human IgM or rabbit anti-human IgA (DAKO, Glostrup, Denmark). Bound antibodies were detected by the conversion of 3,3',5,5'-tetramethylbenzidine (TMB) and, after terminating the reaction by the addition of sulfuric acid, the absorbance was measured at 450 nm. Cut-off values were determined as the mean value plus two times the standard deviation of normal human control sera.

ACPA fine specificity ELISA assays using peptides derived from citrullinated vimentin, fibrinogen and α-enolase were performed as described previously [39,51].

### Statistics

A two-tailed unpaired t-test with a CI of 95% was used to observe differences in reactivity between RA sera and non-RA sera with respect to the citrullinated peptides.

Univariate logistic regression analyses were performed for testing the association between several single risk factors (SE alleles as well as smoking) and anti-citrullinated fibronectin peptide antibodies in early arthritis patients. A Mann-Whitney U test was performed to address associations with the clinical phenotype (HAQ (health assessment questionnaire) score, VAS (visual analog scale) score, swollen joint count and Ritchie index) of the early arthritis patients.

## Results

### Citrullinated fibronectin in synovial fluid samples of RA patients

**Figure 1:**
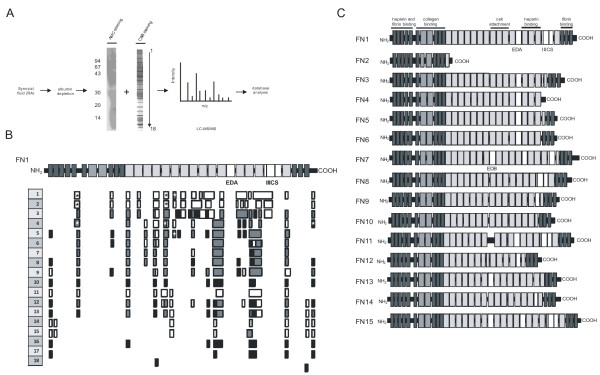
**Identification of (citrullinated) FN in RA synovial fluid**. **A**. RA synovial fluid samples were depleted of albumin and separated by SDS-PAGE and stained with colloidal Coomassie Brilliant Blue. Subsequently, 18 equal slices were excised from the stained gel for both samples. The polypeptides present in the slices were digested with trypsin and analyzed by LC-MS/MS. The presence of citrullinated proteins in the gel was visualized by Western blotting using anti-modified citrulline (AMC) antibodies after modification of the proteins on the blot. The positions of molecular mass markers are indicated on the left (kDa). **B**. The positions of FN peptides detected in RA SF with LC-MS/MS for each of the 18 gel slices of both patient samples are schematically aligned with isoform 1 of fibronectin (FN1). The white bars represent peptides detected in RA1, the black bars peptides detected in RA2 and the grey bars peptides detected in both patients. The positions of the citrullinated residues detected are marked with asterisks. **C**. Schematic overview of the 15 different FN isoforms, resulting from alternative splicing events, documented in the UniProtKB database. The positions of the main alternatively spliced segments EDA, EDB and IIICS are indicated.

To study the immune response to citrullinated FN in RA, first the positions of the citrulline residues in FN isolated from the inflamed joints of RA patients were mapped. One supernatant fraction and one pellet fraction from SF samples obtained from two different RA patients (RA1 and RA2, respectively) were depleted of albumin by a differential precipitation procedure as described previously [49]. This resulted in two pellet fractions for each RA SF sample, which were separated by SDS-PAGE and stained with colloidal Coomassie Brilliant Blue (CBB) (Additional file 1: Figure S1). Subsequently, 18 equal slices, covering the largest polypeptides (slice number 1, molecular weight > 94,000) to the smallest polypeptides (slice number 18, molecular weight < 14,000) were excised from the stained gel for both samples (Figure 1A). The polypeptides present in the slices were digested with trypsin and analyzed by LC-MS/MS. The identity of polypeptides and the positions of citrullinated residues were determined by database searches using Mascot (Version 2.1.03, Matrix Science Inc, Boston, MA, USA). To confirm the presence of citrullinated residues and to distinguish from deamidation of glutamine or asparagine residues, the peptide fragmentation patterns were inspected manually. One of the citrullinated proteins found in the SF of both patients was FN.

Figure 1B shows a schematic overview of all the FN peptide sequences obtained. This indicates that (fragments of) FN were present in material from many gel slices, indicative of a large variety of FN polypeptide lengths in SF samples. FN is a protein for which at least 15 different isoforms exist (Figure 1C); the canonical isoform (FN1) comprises a polypeptide of 2,386 amino acids, in which three repeats can be discerned [52]. FN-derived peptides that were found by these analyses covered most of the FN1 isoform, although for several regions no peptides were detected, which may at least in part be due to poor ionization efficiencies. For one of the alternatively spliced segments, extra domain A (EDA), no peptides were found in the data obtained, whereas several peptides demonstrating the absence of EDA were present. For IIICS, another alternatively spliced region, some peptide sequences were obtained with material from the high molecular weight fractions. Altogether, the mass spectrometry data covered 53% and 28% of the FN isoform 1 sequence for RA1 and RA2, respectively (Figure 1B and Additional file 1: Figure S2).

A search for deiminated arginines identified four citrullinated FN regions containing five citrullinated residues, located at amino acid positions 241, 1035, 1036, 1162 and 2356 (Figure 1B). In RA1, four citrullinated residues were identified (positions 241, 1035, 1036 and 1162), whereas in the second patient (RA2) four citrullinated residues were identified (positions 241, 1035, 1162 and 2356) (Figure 1A and Table 1).

### Anti-citrullinated fibronectin peptide antibodies are specific for RA and are associated with the ACPA response

**Figure 2:**
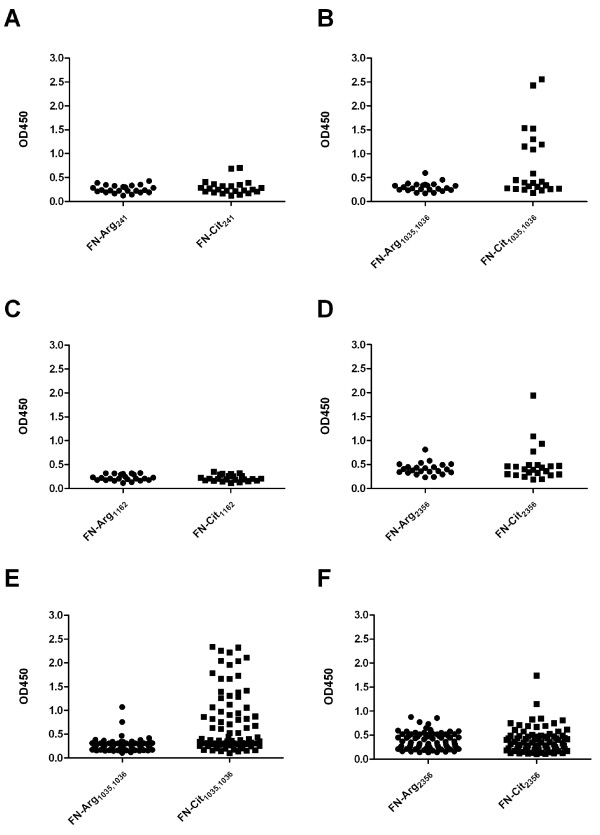
**Recognition of citrullinated fibronectin peptides by RA sera**. The four fibronectin peptide sets (**A**, FN-Arg/Cit_241_; **B**, FN-Arg/Cit_1035,1036_; **C**, FN-Arg/Cit_1162_; **D**, FN-Arg/Cit_2356_) were analyzed by ELISA with 23 sera from established RA patients. Two peptide sets (**E**, FN-Arg/Cit_1035,1036_; **F**, FN-Arg/Cit_2356_) were analyzed with a larger cohort obtained from established RA sera (*n *= 80). OD450 = optical density at 450 nm.

**Figure 3:**
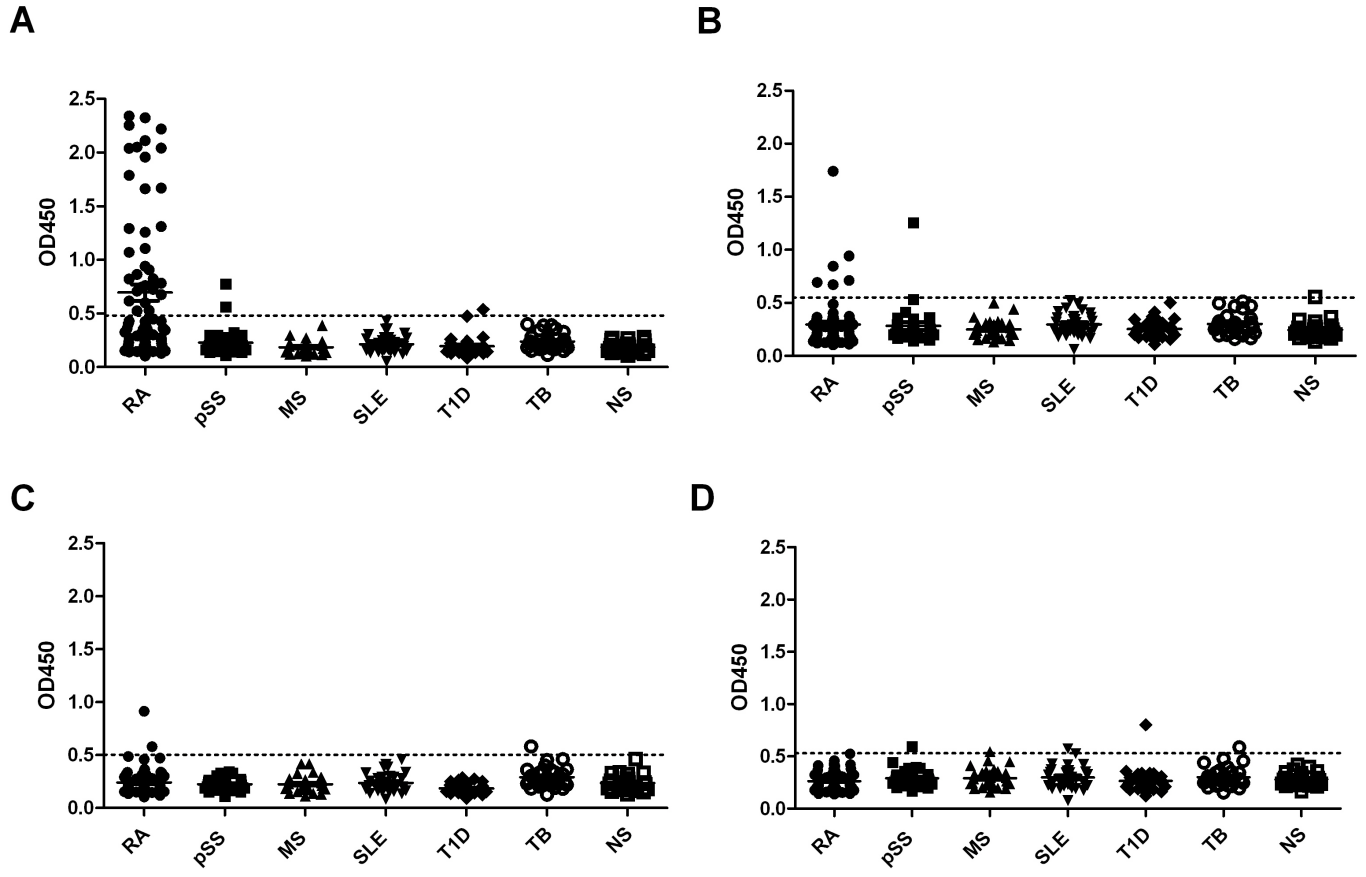
**Recognition of citrullinated fibronectin peptides by diseased and healthy control sera**. The peptides FN-Arg/Cit_1035,1036 _and FN-Arg/Cit_2356 _were used to study the specificity of the anti-FN antibodies. **A**. Reactivity of RA sera and control sera to FN-Cit_1035,1036_. **B**. Reactivity of RA sera and control sera to FN-Cit_2356_. **C**. Reactivity of RA sera and control sera to FN-Arg_1035,1036_. **D**. Reactivity of RA sera and control sera to FN-Arg_2356_. RA (*n *= 75); pSS = primary Sjőgren's syndrome (*n *= 31); MS = multiple sclerosis (*n *= 31); SLE = systemic lupus erythematosus (*n *= 31); T1D = type 1 diabetes (*n *= 32); TB = tuberculosis (*n *= 29); NS = normal human sera (*n *= 32). Broken lines indicate cutoff values (mean + 2*SD of NS data); OD450 = optical density at 450 nm.

To investigate the antigenicity of citrullinated FN, peptides comprising the citrullination sites identified were synthesized, as well as their arginine-containing counterparts (Table 1). A single peptide was synthesized for the flanking citrullination sites at positions 1035 and 1036, similar to the peptide identified in RA SF. The recognition of these peptides by antibodies in established RA patient sera (*n *= 23) was analyzed by ELISA. Two of these peptides, which contained either a citrulline at position 241 or at position 1162 (FN-Cit_241 _and FN-Cit_1162_, respectively), were not recognized by RA sera. In contrast, the other two peptides (FN-Cit_1035,1036 _and FN-Cit_2356_) were reactive with RA sera, and for both this reactivity appeared to be citrulline-dependent (Figure 2A-D). To substantiate these data and to obtain an indication of the frequency by which these peptides are recognized, sera from a second, larger cohort of 80 established RA patients were analyzed. Also these sera were found to be frequently reactive with the FN-Cit_1035,1036 _and FN-Cit_2356 _peptides (Figure 2E, F). Forty-three percent of these sera appeared to recognize FN-Cit_1035,1036_, whereas eight percent was reactive with FN-Cit_2356_. To analyze the disease-specificity of antibodies to these citrullinated FN peptides, sera from 31 multiple sclerosis (MS), 32 type 1 diabetes (T1D), 31 primary Sjőgren's syndrome (pSS), 31 systemic lupus erythematosus (SLE) and 29 tuberculosis (TB) patients, and from 32 healthy individuals, in parallel with 75 established RA sera, were analyzed by ELISA. The results showed that less than two percent of the control sera was reactive with FN-Cit_1035,1036_, whereas one percent displayed reactivity with FN-Cit_2356 _(Figure 3).

**Figure 4:**
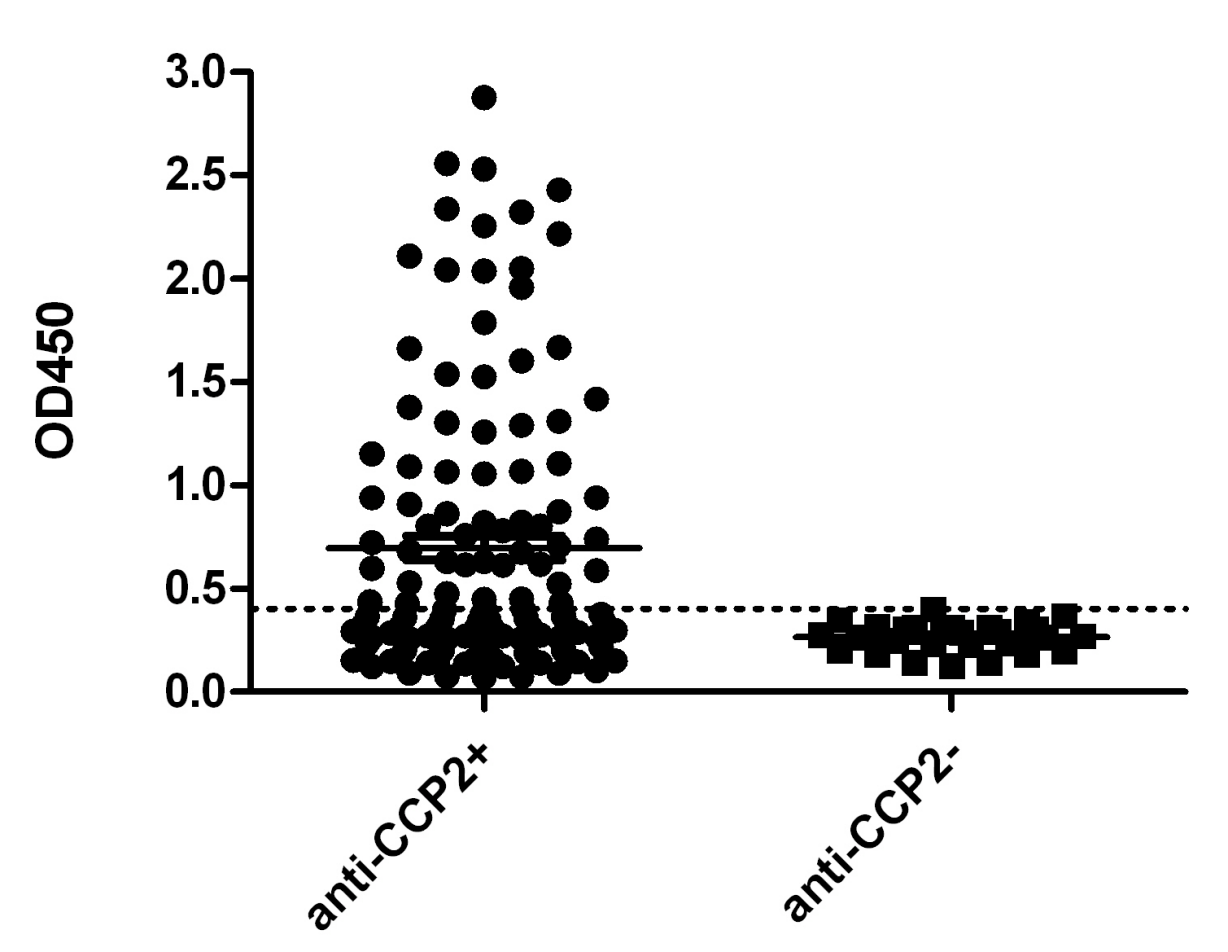
**Anti-citrullinated fibronectin peptide antibodies in relation to anti-CCP2 positivity**. The peptide set FN-Arg/Cit_1035,1036 _was used to study the presence of anti-FN antibodies in anti-CCP2 positive (*n *= 138) and anti-CCP2 negative (*n *= 28) RA sera. The broken line indicates the cut-off value (mean + 2*SD of data obtained with normal human control sera); OD450 = optical density at 450 nm.

Recently, it was shown that RA patients can be divided into two subsets based upon the presence or absence of ACPA in their sera and the CCP2 test appeared to be very suitable to differentiate between these subsets [53]. The analysis of anti FN-Cit_1035,1036 _reactivity in 131 anti-CCP2-positive and 28 anti-CCP2-negative RA sera (Figure 4) showed that the anti-citrullinated fibronectin antibodies were hardly present in anti-CCP2-negative RA sera, corroborating the idea that these antibodies are part of the ACPA response in anti-CCP2-positive RA patients. The combined analyses of the established RA sera resulted in a prevalence of 50 percent for the autoantibodies to peptide FN-Cit_1035,1036 _(Table 2), LTVGLTXXGQPRQY (X represents citrulline), in CCP2-positive established RA sera (*n *= 82)_._

**Table 2:** Sensitivity and specificity of anti-citrullinated fibronectin antibodies.

Patient group	n	anti-FN-Cit_1035,1036 _positive	sensitivity (%)	specificity (%)
**established RA^a^**	82	41	50.0	
**early RA^a^**	278	124	44.6	
				
**Controls**	186	3	1.6	98.4
non-RA	154	3	1.9	98.1
Healthy	32	0	0	100

^a ^Based upon CCP2-positive sera

### Autoantibodies against citrullinated fibronectin peptide are present early in the disease

**Figure 5:**
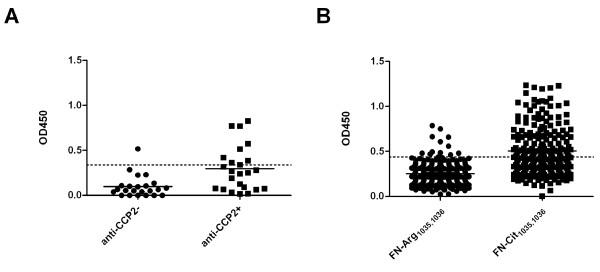
**Anti-citrullinated fibronectin peptide antibodies in early arthritis patient sera**. **A**. Sera from anti-CCP2-negative (*n *= 23) and anti-CCP2-positive (*n *= 24) early arthritis patients were analyzed in the FN-Cit_1035,1036 _ELISA. **B**. Sera from anti-CCP2-positive early arthritis patients (*n *= 278) were analyzed in the FN-Cit_1035,1036 _ELISA. The broken line indicates the cut-off value (mean + 2*SD of data obtained with normal human control sera); OD450 = optical density at 450 nm.

Autoantibodies against CCP2 peptides have been demonstrated to be detectable very early in the disease. Moreover, their presence in pre-disease sera predicts the development of RA [5,6,54]. The diversification of the ACPA response was found to occur mainly in the pre-disease stage. To investigate whether autoantibodies against citrullinated FN are also detectable in the early stages of RA development, a number of sera from early arthritis (EAC) patients (CCP2-negative, *n *= 23; CCP2-positive, *n *= 24) was analyzed. As observed before for the established RA sera, also the EAC sera reactive with the FN-Cit_1035,1036 _peptide represented a subgroup of the anti-CCP2-positive patients (Figure 5A). To substantiate these data and to obtain an indication of the frequency by which the FN-Cit_1035,1036 _peptide was recognized, additional CCP2-positive early arthritis sera (*n *= 278) were analyzed. Forty-five percent of these CCP2-positive EAC sera appeared to be reactive with the citrullinated fibronectin peptide containing citrulline residues at positions 1035 and 1036 (Figure 5B and Table 2). Only a small fraction of these early arthritis sera (4%) displayed some reactivity with the corresponding arginine-containing peptide (Figure 5B).

**Figure 6:**
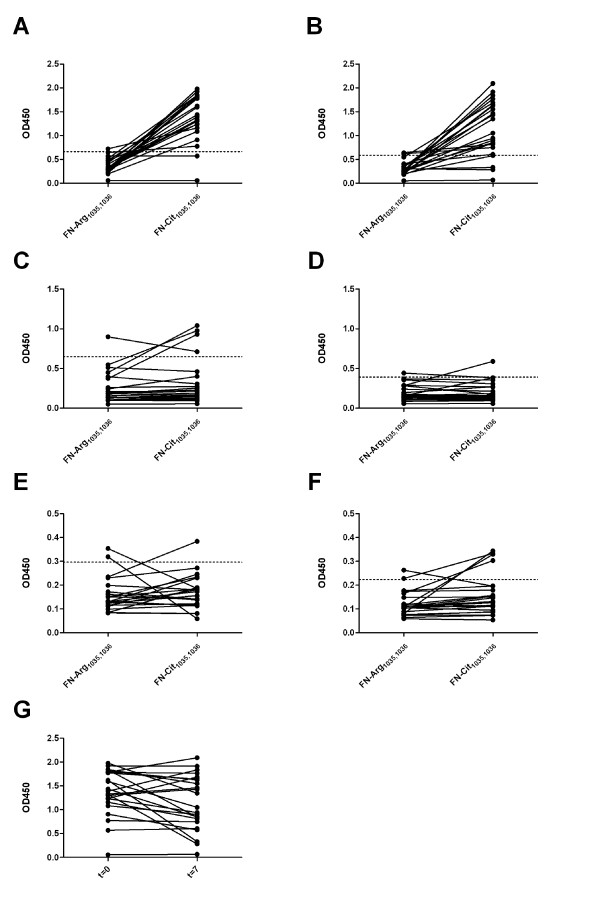
**Anti-FN-Cit_1035,1036 _antibodies are predominantly of the IgG isotype**. Sera from a subset of anti-FN-Cit_1035,1036_-positive early arthritis patients (*n *= 23) taken at baseline (t = 0) and after a median follow-up of seven years later (t = 7) were analyzed for different anti-FN-Cit_1035,1036 _antibody isotypes in ELISA. **A**. IgG isotype reactivity at t = 0. **B**. IgG isotype reactivity at t = 7. **C**. IgM isotype reactivity at t = 0. **D**. IgM isotype reactivity at t = 7. **E**. IgA isotype reactivity at t = 0. **F**. IgA isotype reactivity at t = 7. **G**. Anti-FN-Cit_1035,1036 _IgG reactivity at t = 0 and t = 7. The broken lines indicate the cut-off values determined by normal human control sera.

It is known that after disease onset ACPA isotype switching may occur [55]. To investigate isotype switching of antibodies to citrullinated FN, 23 anti-FN-Cit_1035,1036_-positive sera for which both samples taken at baseline and approximately seven years after disease onset were available, were selected and analyzed in ELISA with isotype-specific (IgG, IgM and IgA) secondary antibody conjugates. At baseline (t = 0), 87% of the reactive sera contained immunoglobulins of the IgG isotype (Figure 6A), whereas 13% contained IgM (Figure 6C) and 4% contained IgA (Figure 6E) type reactivities. After a median follow-up time of seven years (t = 7) the frequency of IgG type antibodies to FN-Cit_1035,1036 _was slightly decreased to 74% (Figure 6B). In these patients the frequency of IgM type anti-FN-Cit_1035,1036 _antibodies decreased to 4% (Figure 6D) In contrast, at this stage the frequency of IgA type antibodies to this citrullinated FN peptide increased to 13% (Figure 6F). All IgM- and IgA-positive patients were also positive for IgG, whereas the simultaneous presence of IgM and IgA reactivities in the same patients was observed in one patient. Except for two patients, in which the IgG type anti-FN-Cit_1035,1036 _antibodies disappeared, the presence of these IgG antibodies did not markedly change in time (Figure 6G).

### Anti-citrullinated fibronectin peptide antibodies are associated with HLA SE alleles

The HLA-DRB1*01, HLA-DRB1*04, HLA-DRB1*10 and HLA-DRB1*14 alleles comprise the group of HLA SE alleles, which are associated with RA [56]. The reactivity to FN-Cit_1035,1036 _was compared with the *HLA-DRB1 *alleles of the 278 early arthritis patients. The results showed that the presence of HLA SE alleles is associated with the production of anti-citrullinated fibronectin antibodies, because patients carrying HLA SE alleles are more than two times more likely to have autoantibodies against citrullinated fibronectin (OR = 2.11; Table 3 and Additional file 1: Table S1). When addressing individual *HLA-DRB1 *alleles, only HLA-DRB1*04 (OR = 1.5), and HLA-DRB1*10 (OR = 1.57) showed a weak to moderate association with the presence of anti-FN-Cit_1035,1036 _antibodies. Although a more pronounced association was observed with HLA-DRB1*08 (OR = 2.45) and HLA-DRB1*16 (OR = 2.43), these data should be interpreted with care because the carriage of these alleles is rare and the data are based on only a low number of patients. Interestingly, a negative association was observed between the presence of anti-citrullinated fibronectin antibodies and two additional *HLA-DRB1 *alleles, HLA-DRB1*09 and HLA-DRB1*11 (OR = 0.19 and OR = 0.41, respectively) (Additional file 1: Table S1).

**Table 3:** Association of anti-citrullinated fibronectin antibodies with smoking and SE alleles

		Anti-cit FN neg. (%)^d^	Anti-cit FN pos. (%)^d^	OR (95% CI)
**Smoking^a^**	-	63 (46.7)	40 (38.1)	**1.42 (0.85 to 2.39)**
	+	72 (53.3)	65 (61.9)	
**HLA SE alleles^b^**	-	39 (26.5)	57 (14.6)	**2.11 (1.13 to 3.92)**
	+	108 (73.5)	213 (85.4)	

^a ^Based upon information obtained from 240 RA patients. ^b ^Based upon information obtained from 270 RA patients. 95% CI, 95% confidence interval; OR, odds ratio

In addition, the potential relationship of smoking with the production of anti-citrullinated fibronectin antibodies was assessed and a weak association was found (OR = 1.42). We did not observe significant associations between the presence of anti-citrullinated fibronectin antibodies and clinical parameters, such as VAS score, HAQ score, Ritchie index or swollen joint count at baseline (data not shown).

## Discussion

The analysis of citrullinated proteins in the synovial fluids of two rheumatoid arthritis patients revealed fibronectin as one of the multiply citrullinated proteins in both patients. Two of the four synthetic peptides that were derived from the citrullinated regions of FN appeared to be reactive with ACPA in the sera of RA patients. The most frequently targeted FN peptide, FN-Cit_1035,1036_, which contains two adjacent citrullines, was recognized by 50% of established and 45% of early ACPA-positive RA patients. Like ACPA in general, anti-FN-Cit_1035,1036 _antibodies appeared to be associated with *HLA-DRB1 *shared epitope alleles.

FN is a complex protein, characterized by the presence of three types of repeats in its polypeptide sequence, for which many isoforms resulting from alternative splicing events have been described (Figure 1C). The UniProtKB database provides details of 15 isoforms, with isoform 1 (FN1 in Figure 1C) as the canonical sequence. The overall sequence coverage of the mass spectrometry datasets relative to FN1 comprises 53% and 28% for both synovial fluid samples, respectively. It is likely that multiple FN isoforms are expressed in the inflamed synovia of RA patients and our data do not allow drawing conclusions on their relative abundance. Three regions of FN are especially prone to alternative splicing; at the protein level these are termed extra domain A (EDA), extra domain B (EDB) and type III connecting segment (IIICS). Although peptides were found that match part of the IIICS sequence, no peptides were detected for EDA and EDB. Moreover, a few peptides provide evidence for the absence of EDA and EDB in the isoform(s) they originate from, because their sequences covered the regions immediately N- and C-terminal from EDA or from EDB. The EDA domain, also designated EIIIA, has been implicated in inflammation, because it was shown to be involved in Toll-like receptor (TLR) 4 activation [57]. Although our data do not support the presence of EDA-containing FN isoforms in the synovial fluid of RA patients, it has been demonstrated previously that EDA-containing FN is produced in the RA synovium and is expressed abundantly in RA SF [58,59]. Moreover, recently Lefebvre and colleagues showed that EDA-containing FN stimulated leukotriene synthesis and neutrophil recruitment via TLR activation in a mouse model [60]. Although we could not detect any peptides in the EDA region, our data do not exclude the presence of EDA-containing FN isoforms in RA synovial fluid samples, because we have only analyzed a limited number of patients or because of technical limitations, such as ionization efficiencies.

The interpretation of the data is further complicated by the fact that FN-derived peptides were found in material from many gel slices, which indicates a high heterogeneity of FN polypeptide lengths. It is likely that this is at least in part due to the presence of proteolytic enzymes in the synovial fluid from inflamed joints, which cleave the FN polypeptides in different fragments. It is known that during inflammation proteases are active in synovial fluid and can contribute to RA pathogenesis [61,62]. Indeed, the fragmentation of FN in inflammatory SF [63], as well as in cartilage of RA and osteoarthritis patients [64] has been reported previously.

Our analyses identified five citrullinated residues in FN from RA patient SF. Except for two adjacent citrullines, these residues are found in distant regions of the FN polypeptide chain. Our data do not provide information on the extent to which these residues are citrullinated in RA SF and it is likely that differences between patients exist. This is substantiated by the observation that in material from RA2 for the region containing the two adjacent citrullines only one of these two residues (1035) was found to be citrullinated, whereas both were citrullinated in RA1. Two other studies have previously reported the presence of citrullinated FN in SF and synovial tissue of RA patients [41,43]. However, these studies did not reveal the positions of the citrullinated residues. Our data do not exclude the possibility that FN is also citrullinated at other positions in RA SF, because material from only two patients was analyzed in detail and the sequence coverage was not more than 53%. Moreover, if citrullination enhances FN's susceptibility to proteolytic cleavage, citrullinated peptides may have escaped detection as a result of cleavages by endogenous proteases that cleave the polypeptide close to the citrullinated residue.

A synthetic peptide approach to investigate the recognition of citrullinated epitopes of FN by RA autoantibodies revealed that the major autoepitope is located in the region containing the two adjacent citrullines (amino acids 1035 and 1036). Only one of the other three citrullinated peptides was recognized by some RA sera. The fact that RA sera were only reactive with two of these peptides is consistent with the results of other studies showing that the amino acids flanking the citrulline residue contribute to the formation of auto-epitopes [1]. Several studies [24,34,65] with synthetic citrullinated peptides (derived from vimentin, fibrinogen and α-enolase) showed that not all peptides containing citrullinated residues are recognized by patient sera, indicating that not only the citrulline is important, but also the amino acids surrounding the citrullinated residue. However, it should be noted that the use of synthetic peptides in general does not allow the identification of reactivities with conformational and/or discontinuous epitopes. Therefore, our data do not exclude the possibility that the citrullinated residues identified are part of conformational epitopes and that autoantibodies to these epitopes may be present in RA patient sera. Although autoantibodies to FN have been detected in RA as well as SLE patients before [66,67], our data are the first to describe antibodies that target FN in a citrulline-dependent manner. The autoantibodies that can be detected with the FN-Cit_1035,1036 _peptide represent a subset of ACPA, which is substantiated by the lack of correlation between the levels of reactivity with CCP2 and with FN-Cit_1035,1036 _(Additional file 1: Figure S3). In total, 50% of the (anti-CCP2-positive) established RA patients showed reactivity to FN-Cit_1035,1036_, compared to two percent of the controls (non-RA and healthy individuals; Table 2).

**Figure 7:**
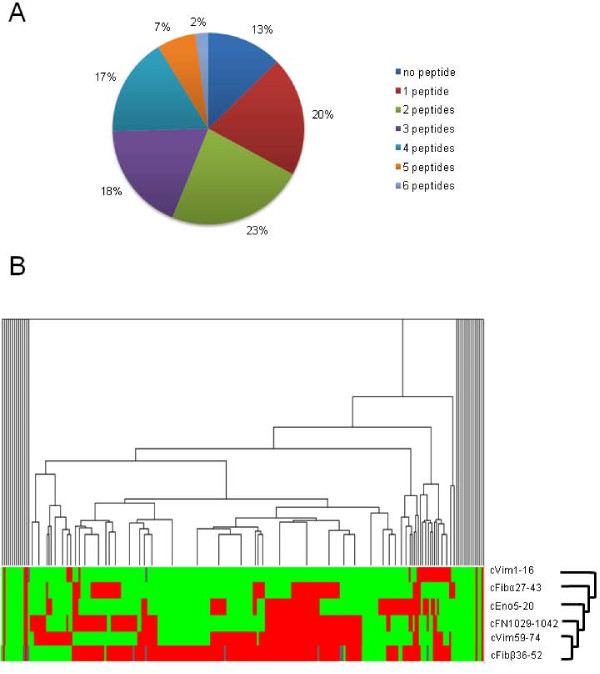
**Anti-FN-Cit_1035,1036 _in relation to other ACPA**. The reactivity of EAC sera (*n *= 228) with FN-Cit_1035,1036 _was compared with the presence of antibodies to other citrullinated peptides, which are derived from vimentin (1 to 16: STCitSVSSSSYCitCitMFGG and 59 to 74: VYATCitSSAVCitLCitSSVP), fibrinogen (α-fibrinogen 27 to 43: FLAEGGGVCitGPRVVERH; β-fibrinogen 36 to 52: NEEGFFSACitGHRPLDKK) and α-enolase (5 to 20: KIHACitEIFDSCitGNPTV) [39]. **A**. Fraction of patients recognizing 0 to 6 citrullinated peptides. **B**. Heat map showing the presence of antibodies to the citrullinated peptides obtained by an unsupervised cluster analysis. Red and green mark positive and negative sera, respectively. Missing values are depicted in grey.

The possibility existed that the anti-FN-Cit_1035,1036 _subset of ACPA overlapped with another subset that has been identified previously, as a result of epitope similarities. ACPA fine-specificity data were available for most of the EAC patient sera. The reactivity of these sera with FN-Cit_1035,1036 _was compared with their reactivity with two citrullinated peptides derived from vimentin (vimentin-1-16: STCitSVSSSSYCitCitMFGG; vimentin-59-74: VYATCitSSAVCitLCitSSVP), two peptides derived from fibrinogen (α-fibrinogen-27-43: FLAEGGGVCitGPRVVERH; β-fibrinogen-36-52: NEEGFFSACitGHRPLDKK) and one peptide derived from α-enolase (α-enolase-5-20: KIHACitEIFDSCitGNPTV) [39]. These analyses showed that most of the EAC sera recognized multiple citrullinated epitopes (Figure 7A). A large overlap of anti-FN-Cit_1035,1036 _with reactivities to any of the other peptides was observed, as might be expected from previous data [26,68]. However, some sera appeared to react exclusively with FN-Cit_1035,1036 _and not with the other citrullinated epitopes (Figure 7B). These data are underscoring the large heterogeneity of the ACPA response in RA and indicate that the anti-FN-Cit_1035,1036 _antibodies are one of the most abundant ACPA subclasses that can be detected with synthetic peptides derived from citrullinated synovial proteins. Taken together, these data indicate that this citrullinated FN peptide may be used to investigate the fine-specificity of ACPA in more detail and may be complementary to other citrullinated molecules in ACPA profiling.

The anti-FN-Cit_1035,1036 _antibodies present in early arthritis patients appeared to be predominantly of the IgG isotype. Only a small percentage of sera was found to contain IgM or IgA type anti-FN-Cit_1035,1036 _antibodies. After a median follow-up of seven years, the IgG type reactivities were hardly changed, whereas the prevalence of IgM reactivity against FN-Cit_1035,1036 _was decreased, and that of IgA was somewhat increased. Similar observations have been reported for samples from undifferentiated arthritis patients (who developed RA) taken either at baseline or after one year follow-up [36]. It remains an open question whether the anti-citrullinated FN antibodies play a pathophysiological role. FN-containing immune complexes are likely to be formed in the inflamed joints of RA patients and this may occur already early during disease development. Further studies will be required to elucidate whether citrullinated FN is involved in the inflammatory process.

The presence of anti-citrullinated FN antibodies in the early arthritis patients was associated with HLA SE alleles (OR = 2.11). Of the individual SE alleles, only HLA-DRB1*04 and HLA-DRB1*10 showed a weak association with the presence of anti-citrullinated FN peptide antibodies (Additional file 1: Table S1). Previously, Snir and co-workers demonstrated that antibodies against multiple citrullinated antigens (for example, vimentin, fibrinogen, α-enolase and the C1-epitope of type II collagen) were associated with SE alleles, particularly with HLA-DRB1*04 [26,69]. Our data show a negative association of HLA-DRB1*09 and HLA-DRB1*11 with the presence of anti-citrullinated FN peptide antibodies (OR = 0.19 and OR = 0.41, respectively). This may be in agreement with the previously reported association of the HLA-DRB1*0901 haplotype with reduced levels of anti-CCP antibodies [70]. Paradoxically, HLA-DRB1*09 has been reported previously to be associated with RA in Asian as well as Caucasian individuals [71,72]. In addition, it should be noted that, because ACPA-positive RA patients comprise a strong prevalence for SE-allelles, other *HLA-DRB1 *alleles are less frequently present than SE-alleles and, therefore, might seem to be protective. As a consequence, the negative association observed with HLA-DRB1*09 and HLA-DRB1*11 might also be the result of skewing [73].

We did not detect a significant association between clinical phenotype and the presence of anti-citrullinated fibronectin in ACPA-positive RA patients. Recently, Scherer and colleagues also observed no effect on radiographic joint damage in patients that were positive for several citrullinated epitopes [37]. Nevertheless, a weak correlation between the presence of autoantibodies against citrullinated FN and smoking, a risk factor for ACPA generation, particularly in individuals that carry the SE alleles [16,17], was observed.

## Conclusion

Five citrullinated residues were identified in fibronectin isolated from the inflamed joints of RA patients. An epitope containing two adjacent citrullines at positions corresponding to residues 1035 and 1036 appeared to be most frequently recognized by RA sera. Our data not only show that antibodies against citrullinated FN are present in RA patients, but also demonstrate that the anti-FN antibodies represent a subgroup of anti-CCP2 antibodies and that they can already be detected very early in the disease. Moreover, anti-FN-Cit_1035,1036 _antibodies are associated primarily with HLA SE alleles.

## Abbreviations

ACPA: anti-citrullinated protein antibodies; AMC. anti-modified citrulline; BSA: bovine serum albumin; CBB: Coomassie Brilliant Blue; CCP: cyclic citrullinated peptide; CI: coincidence interval; EAC: early arthritis clinic; EDA: extra domain A; EDB: extra domain B; FN: fibronectin; HAQ: health assessment questionnaire; HLA: human leukocyte antigen; IIICS: type III connecting segment; LC-MS/MS: liquid chromatography - tandem mass spectrometry; LTQ: linear trap quadrupole; MS: multiple sclerosis; NS: normal sera; OR: odds ratio; PAD: peptidylarginine deiminase; PBS: phosphate-buffered saline; pSS: primary Sjogren's syndrome; RA: rheumatoid arthritis; RF: rheumatoid factor; SE: shared epitope; SF: synovial fluid; SLE: systemic lupus erythematosus; T1D: type 1 diabetes; TB: tuberculosis; TLR: Toll-like receptor; TMB: 3,3',5,5'-tetramethylbenzidine; VAS: visual analog scale

## Competing interests

The authors declare that they have no competing interests.

## Authors' contributions

JvB carried out the proteomic studies and part of the immunoassays, and participated in design of the study, interpretation of the data and drafting of the manuscript. AW participated in the analysis and interpretation of the data, performed the statistical analysis and contributed to the preparation of the manuscript. JS-V performed part of the immunoassays. JD participated in the design of the study, generated the synthetic peptides and participated in the preparation of the manuscript. RT participated in the design of the study, the interpretation of the data and the preparation of the manuscript. GP conceived of the study, participated in its design and coordination, contributed to the interpretation of the data and helped to draft the manuscript. All authors read and approved the final manuscript.

## Supplementary Material

Additional file 1 **Supplementary Table 1 and Supplementary Figures 1 to 3**. Supplementary Table 1: Association of anti-citrullinated fibronectin antibodies and HLA-DRB1 alleles in anti-CCP2-positive early arthritis patients. Supplementary Figure 1: Overview of the handling of synovial fluid samples. Supplementary Figure 2: Fibronectin-derived peptides identified in the synovial fluid of RA patients. Supplementary Figure 3: Correlation between anti-CCP2 and anti-FN-Cit_1035,1036 _reactivities. Click here for file
