# Supplementary material for: Anti-citrullinated fibronectin antibodies in rheumatoid arthritis are associated with human leukocyte antigen-DRB1 shared epitope alleles
Source: Arthritis Res Ther. 2012 Feb 17;14(1):R35. doi: 10.1186/ar3744 (PMC3392834; doi:10.1186/ar3744)

Supplementary material

Anti-citrullinated fibronectin antibodies in rheumatoid arthritis are associated with HLA-DRB1 shared epitope alleles.

Joyce J.B.C. van Beers, Annemiek Willemze, Judith Stammen-Vogelzangs, Jan W. Drijfhout, Rene E.M. Toes and Ger J.M. Pruijn

SUPPLEMENTARY TABLES

Table 1. Association of anti-citrullinated fibronectin antibodies and HLA-DRB1 alleles in anti-CCP2-positive early arthritis patients.

		Anti-cit FN neg. (%)[d]	Anti-cit FN pos. (%)[d]	OR (95% CI)
**Smoking[a]**	-	63 (46.7)	40 (38.1)	**1.42 (0.85-2.39)**
	+	72 (53.3)	65 (61.9)	
**HLA SE alleles[b]**	-	39 (26.5)	57 (14.6)	**2.11 (1.13-3.92)**
	+	108 (73.5)	213 (85.4)	
**HLA-DRB1*01[c]**	-	112 (75.2)	93 (75.0)	1.01 (0.58-1.75)
	+	37 (24.8)	31 (25.0)	
**HLA-DRB1*03[c]**	-	118 (79.2)	105 (84.7)	0.69 (0.37-1.29)
	+	31 (20.8)	19 (15.3)	
**HLA-DRB1*04[c]**	-	70 (47.0)	46 (37.1)	**1.5 (0.92-2.44)**
	+	79 (53.0)	78 (62.9)	
**HLA-DRB1*07[c]**	-	131 (87.9)	110 (88.7)	0.93 (0.44-1.95)
	+	18 (12.1)	14 (11.3)	
**HLA-DRB1*08[c]**	-	147 (98.7)	120 (96.8)	2.45 (0.44-13.61)
	+	2 (1.3)	4 (3.2)	
**HLA-DRB1*09[c]**	-	137 (91.9)	122 (98.4)	**0.19 (0.04-0.85)**
	+	12 (8.1)	2 (1.6)	
**HLA-DRB1*10[c]**	-	137 (91.9)	109 (87.9)	**1.57 (0.71-3.5)**
	+	12 (8.1)	15 (12.1)	
**HLA-DRB1*11[c]**	-	125 (83.9)	115 (92.7)	**0.41 (0.18-0.91)**
	+	24 (16.1)	9 (7.3)	
**HLA-DRB1*12[c]**	-	145 (97.3)	121 (97.6)	0.9 (0.20-4.09)
	+	4 (2.7)	3 (2.4)	
**HLA-DRB1-13[c]**	-	129 (86.6)	113 (91.1)	0.63 (0.29-1.37)
	+	20 (13.4)	11 (8.9)	
**HLA-DRB1-14[c]**	-	144 (96.6)	120 (96.8)	0.96 (0.25-3.66)
	+	5 (3.4)	4 (3.2)	
**HLA-DRB1*15[c]**	-	113 (75.8)	91 (73.4)	1.14 (0.66-1.97)
	+	36 (24.2)	33 (26.6)	
**HLA-DRB1*16[c]**	-	148 (99.3)	122 (98.4)	2.43 (0.22-27.08)
	+	1 (0.7)	2 (1.6)	

[a] Based upon information obtained from 240 RA patients.
[b] Based upon information obtained from 270 RA patients.
[c] Based upon information obtained from 278 RA patients.
[d] Values are the number and percentages of early arthritis patients (EAC) negative or positive (cut-off = mean + 2*SD) for anti-FN-Cit$_{1035,1036}$ antibodies.
OR = odds ratio; 95% CI = 95% confidence interval.

SUPPLEMENTARY FIGURES

Supplementary Figure 1. Overview of the handling of synovial fluid samples

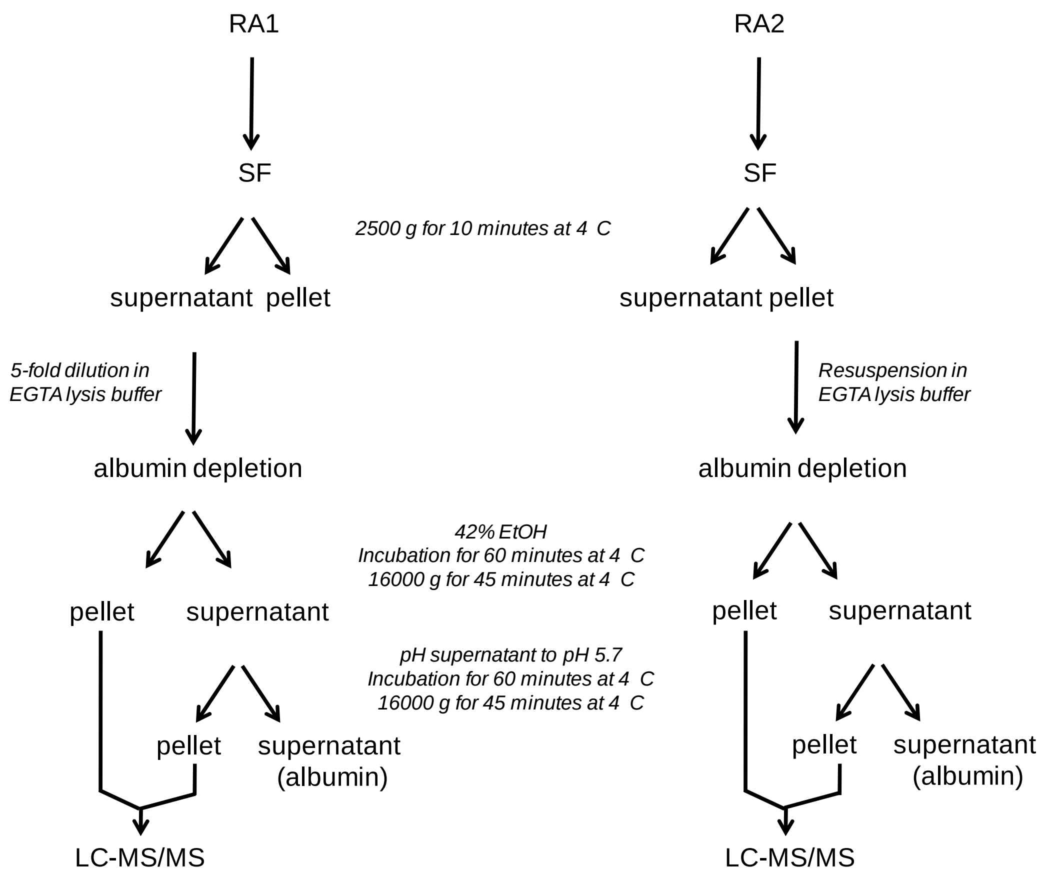

Supplementary Figure 2. Fibronectin-derived peptides identified in the synovial fluid of RA patients.

The fibronectin peptides detected in synovial fluid samples of RA patients are indicated by lines above and below the amino acid sequence of human fibronectin (for RA1 and RA2, respectively) and the citrullinated residues present in these peptides are marked with a grey box. The extra domain A (EDA) covers the region bordered by the amino acids 1631 and 1721. In one peptide amino acid 1630 was fused to amino acid 1722 (marked by asterisks).

```
   1 MLRGPGPGLL LLAVQCLGTA VPSTGASKSK RQAQQMVQPQ SPVAVSQSKP GCYDNGKHYQ INQQWERTYL GNALVCTCYG GSRGFNCESK
  91 PEAEETCFDK YTGNTYRVGD TYERPKDSMI WDCTCIGAGR GRISCTIANR CHEGGQSYKI GDTWRRPHET GGYMLECVCL GNGKGEWTCK
 181 PIAEKCFDHA AGTSYVVGET WEKPYQGWMM VDCTCLGEGS GRITCTSRNR CNDQDTRTSY RIGDTWSKKD NRGNLLQCIC TGNGRGEWKC
 271 ERHTSVQTTS SGSGPFTDVR AAVYQPQPHP QPPPYGHCVT DSGVVYSVGM QWLKTQGNKQ MLCTCLGNGV SCQETAVTQT YGGNSNGEPC
 361 VLPFTYNGRT FYSCTTEGRQ DGHLWCSTTS NYEQDQKYSF CTDHTVLVQT RGGNSNGALC HFPFLYNNHN YTDCTSEGRR DNMKWCGTTQ
 451 NYDADQKFGF CPMAAHEEIC TTNEGVMYRI GDQWDKQHDM GHMMRCTCVG NGRGEWTCIA YSQLRDQCIV DDITYNVNDT FHKRHEEGHM
 541 LNCTCFGQGR GRWKCDPVDQ CQDSETGTFY QIGDSWEKYV HGVRYQCYCY GRGIGEWHCQ PLQTYPSSSG PVEVFITETP SQPNSHPIQW
 631 NAPQPSHISK YILRWRPKNS VGRWKEATIP GHLNSYTIKG LKPGVVYEGQ LISIQQYGHQ EVTRFDFTTT STSTPVTSNT VTGETTPFSP
 721 LVATSESVTE ITASSFVVSW VSASDTVSGF RVEYELSEEG DEPQYLDLPS TATSVNIPDL LPGRKYIVNV YQISEDGEQS LILSTSQTTA
 811 PDAPPDPTVD QVDDTSIVVR WSRPQAPITG YRIVYSPSVE GSSTELNLPE TANSVTLSDL QPGVQYNITI YAVEENQEST PVVIQQETTG
 901 TPRSDTVPSP RDLQFVEVTD VKVTIMWTPP ESAVTGYRVD VIPVNLPGEH GQRLPISRNT FAEVTGLSPG VTYYFKVFAV SHGRESKPLT
 991 AQQTTKLDAP TNLQFVNETD STVLVRWTPP RAQITGYRLT VGLTRRGQPR QYNVGPSVSK YPLRNLQPAS EYTVSLVAIK GNQESPKATG
1081 VFTTLQPGSS IPPYNTEVTE TTIVITWTPA PRIGFKLGVR PSQGGEAPRE VTSDSGSIVV SGLTPGVEYV YTIQVLRDGQ ERDAPIVNKV
1171 VTPLSPPTNL HLEANPDTGV LTVSWERSTT PDITGYRITT TPTNGQQGNS LEEVVHADQS SCTFDNLSPG LEYNVSVYTV KDDKESVPIS
1261 DTIIPAVPPP TDLRFTNIGP DTMRVTWAPP PSIDLTNFLV RYSPVKNEED VAELSISPSD NAVVLTNLLP GTEYVVSVSS VYEQHESTPL
1351 RGRQKTGLDS PTGIDFSDIT ANSFTVHWIA PRATITGYRI RHHPEHFSGR PREDRVPHSR NSITLTNLTP GTEYVVSIVA LNGREESPLL
1441 IGQQSTVSDV PRDLEVVAAT PTSLLISWDA PAVTVRYYRI TYGETGGNSP VQEFTVPGSK STATISGLKP GVDYTITVYA VTGRGDSPAS
1531 SKPISINYRT EIDKPSQMQV TDVQDNSISV KWLPSSSPVT GYRVTTTPKN GPGPTKTKTA GPDQTEMTIE GLQPTVEYVV SVYAQNPSGE
1621 SQPLVQTAVT NIDRPKGLAF TDVDVDSIKI AWESPQGQVS RYRVTYSSPE DGIHELFPAP DGEEDTAELQ GLRPGSEYTV SVVALHDDME
1711 SQPLIGTQST AIPAPTDLKF TQVTPTSLSA QWTPPNVQLT GYRVRVTPKE KTGPMKEINL APDSSSVVVS GLMVATKYEV SVYALKDTLT
1801 SRPAQGVVTT LENVSPPRRA RVTDATETTI TISWRTKTET ITGFQVDAVP ANGQTPIQRT IKPDVRSYTI TGLQPGTDYK IYLYTLNDNA
1891 RSSPVVIDAS TAIDAPSNLR FLATTPNSLL VSWQPPRARI TGYIIKYEKP GSPPREVVPR PRPGVTEATI TGLEPGTEYT IYVIALKNNQ
1981 KSEPLIGRKK TDELPQLVTL PHPNLHGPEI LDVPSTVQKT PFVTHPGYDT GNGIQLPGTS GQQPSVGQQM IFEEHGFRRT TPPTTATPIR
2071 HRPRPYPPNV GEEIQIGHIP REDVDYHLYP HGPGLNPNAS TGQEALSQTT ISWAPFQDTS EYIISCHPVG TDEEPLQFRV PGTSTSATLT
2161 GLTRGATYNI IVEALKDQQR HKVREEVVTV GNSVNEGLNQ PTDDSCFDPY TVSHYAVGDE WERMSESGFK LLCQCLGFGS GHFRCDSSRW
2251 CHDNGVNYKI GEKWDRQGEN GQMMSCTCLG NGKGEFKCDP HEATCYDDGK TYHVGEQWQK EYLGAICSCT CFGGQRGWRC DNCRRPGGEP
2341 SPEGTTGQSY NQYSQRYHQR TNTNVNCPIE CFMPLDVQAD REDSRE
```

Supplementary Figure 3. Correlation between anti-CCP2 and anti-FN-Cit$_{1035,1036}$ reactivities.

Correlation diagram showing the levels of anti-FN-Cit$_{1035,1036}$ and anti-CCP2 reactivities of (established) RA sera (n=110). Broken lines represent the cut-off values. OD450 = optical density at 450 nm.

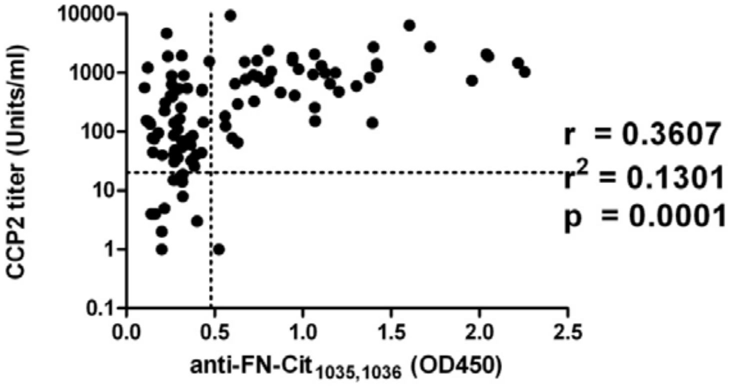

Supplement: Additional file 1 — Supplementary Table 1 and Supplementary Figures 1 to 3. Supplementary Table 1: Association of anti-citrullinated fibronectin antibodies and HLA-DRB1 alleles in anti-CCP2-positive early arthritis patients. Supplementary Figure 1: Overview of the handling of synovial fluid samples. Supplementary Figure 2: Fibronectin-derived peptides identified in the synovial fluid of RA patients. Supplementary Figure 3: Correlation between anti-CCP2 and anti-FN-Cit1035,1036 reactivities. [file ar3744-S1.PDF]
